# Evaluation of lyophilized royal jelly and garlic extract emulgels using a murine model infected with methicillin-resistant *Staphylococcus aureus*

**DOI:** 10.1186/s13568-022-01378-x

**Published:** 2022-03-21

**Authors:** Mona H. El-Gayar, Rania A. H. Ishak, Ahmed Esmat, Mohammad M. Aboulwafa, Khaled M. Aboshanab

**Affiliations:** 1 Department of Microbiology & Immunology, Faculty of Pharmacy, Ain Shams University, Organization of African Unity St., Abbassia, Cairo, 11566 Egypt; 2 Department of Pharmaceutics and Industrial Pharmacy, Faculty of Pharmacy, Ain Shams University, Cairo, 11566 Egypt; 3 Department of Pharmacology and Toxicology, Faculty of Pharmacy, Ain Shams University, Cairo, 11566 Egypt; 4 Faculty of Pharmacy, King Salman International University, Ras‑Sedr, South Sinai Egypt

**Keywords:** In-vivo, Lyophilized royal jelly, Garlic extract, Emulgels, MRSA

## Abstract

The limited therapeutic options associated with methicillin-resistant *Staphylococcus aureus* (MRSA) necessitate search for innovative strategies particularly, use of natural extracts such as lyophilized royal jelly (LRJ) and garlic extract (GE). Therefore, out study aimed to formulate emulgels containing different concentrations of both LRJ and GE and to evaluate their activities using a murine model infected with MRSA clinical isolate. Four plain emulgel formulas were prepared by mixing stearic acid/yellow soft paraffin-based O/W emulsion formulae based on Carbopol 940, Na alginate, Na carboxymethylcellulose or Hydroxypropyl methyl cellulose E4. Sodium alginate-based emulgel was selected for preparation of four medicated emulgel formulations combining LRJ and GE at four different concentrations. The selected medicated emulgels were used for the in vivo studies. The emulgel formulated with Na alginate and HPMC (MF_3_) exhibited optimum smooth homogeneous consistency, neutral pH, acceptable viscosity, spreadability, extrudability values and best storage stability properties. In vivo results revealed that, the wounds infected with MRSA isolate in rates were wet (oozing) and showed pus formation when compared to injured uninfected wounds. MF_3_ formula containing 4% LRJ and 50% GE showed the maximum wound healing properties, both in the apparent physical wound healing measurements and in the histopathological examination. In conclusion, the medicated emulgel formulation (MF_3_) prepared with Na alginate was found optimum for topical application. MF_3_ formula containing 4% LRJ and 50% GE has shown the highest in vivo wound healing capacities. Further clinical studies should be conducted to prove both its safety and efficacy and the potential use in human.

## Key points

1. Four plain emulgel formulas were prepared and investigated for physiochemical and stability properties (F1–F4).
2. The emulgel F_3_ exhibited optimum smooth homogeneous consistency, neutral pH, acceptable viscosity, spreadability, extrudability values and best storage stability properties.
3. A murine skin model infected with clinical MRSA isolate was established and wounds were monitored via apparent physical wound healing measurements and histopathological examination.
4. The MF3 containing 4% LRJ and 50% GE has shown the highest in vivo wound healing capacities.

## Introduction

Methicillin-resistant *Staphylococcus aureus* (MRSA) is one of the most common causes of nosocomial and community-acquired infections with limited therapeutic options (Siddiqui and Koirala 2021). According to World Health Organization (WHO) reports, MRSA was one of the top published lists of 12 pathogens that pose public health threat (Reardon 2014) requiring global concern for discovering of new antibacterial agents and development of new control strategies (Duval et al. 2019; Tong et al. 2016; Turner et al. 2019). Targeting MRSA virulence such as biofilm, adhesion and tissue colonization are considered one of innovative strategies to combat its pathogenesis and prevent complications (Marra 2004; Donlan and Costerton 2002; Foster et al. 2014).Various in-vitro and in-vivo studies had documented the use of natural crude extracts of garlic and royal jelly (RJ) as anti-virulence agents for controlling MRSA infections (El-Gayar et al. 2016; Lin et al. 2020; Zhu and Zeng 2020; Nidadavolu et al. 2012). Further studies were conducted and showed that both allicin and ajoene are the two major active ingredients of the GE having antibiofilm, (Tsao et al. 2003; Girish et al. 2019), antimicrobial, (Li et al. 2015; Yadav et al. 2015; Fratianni et al. 2016) and antifungal properties (Li et al. 2015, 2016; Fratianni et al. 2016; Wr et al. 2014). Moreover, honey containing RJ and RJ as crude extracts have been investigated in the past few decades and their antimicrobial and wound healing properties have been confirmed (El-Gayar et al. 2016; Lin et al. 2020; Zhu and Zeng 2020; Nidadavolu et al. 2012; Boukraâ and Sulaiman 2009; Dinkov et al. 2016). Their activities against multidrug resistance (MDR) pathogens such as methicillin-resistant MRSA and vancomycin-resistant *Enterococcus* (VRE) have been laboratory confirmed (El-Gayar et al. 2016; Dinkov et al. 2016; Laid 2015; Cong et al. 2019; Brudzynski et al. 2015). Another study reported that RJ did not inhibit MRSA infection in an experimental animal model however, it was able to decrease severity of infection remarkably when applied to the site of infection at regular bases (Gunaldi et al. 2014). All previously mentioned studies were based on the use the crude extracts of either RJ or the GE for investigating their biological activities (El-Gayar et al. 2016; Lin et al. 2020; Zhu and Zeng 2020; Nidadavolu et al. 2012; Boukraâ and Sulaiman 2009; Dinkov et al. 2016). However, till now no studies were conducted for pharmaceutical preparation of suitable topical preparations containing these biologically active natural products. Therefore, this study aimed to formulate and test stability and pharmaceutical properties of different emulgels containing various concentrations of both lyophilized RJ (LRJ) and GE and to evaluate their activities to abrogate the infection of MRSA using a superficial skin infection murine model.

## Materials and methods

### Natural products and chemicals

LRJ was kindly supplied from Egyptian International Pharmaceutical Industries (EIPICO^®^, Cairo, Egypt). Aqueous GE was prepared by filtering extracted chopped raw garlic in deionized water. Stearic acid, soft yellow paraffin, propylene glycol, and sodium carboxymethyl cellulose (Na CMC) were obtained from El-Nasr Pharmaceuticals (Cairo, Egypt). Butylated hydroxytoluene (BHT), methyl paraben, propyl paraben, triethanolamine (TEA), Carbopol 940 were gifted from ATCO^®^ Pharma for Pharmaceutical Industries (El-Menofia, Egypt). Sodium alginate (Na alginate), S-allyl-l-cysteine (SAC), and Royal Jelly acid (RJA) were purchased from Merck^®^, Darmstadt, Germany. Hydroxypropyl methyl cellulose (HPMC E4) has been gotten from Memphis^®^ Pharmaceutical Co. (Cairo, Egypt). Deionized water was produced from a Milli-Q Gradient A10 System (Millipore, Billerica, MA. USA).

### MRSA clinical isolate

MRSA clinical isolate coded CCASU-MRSA3 was obtained from the Culture Collection Ain Shams University (CCASU-MRSA3) and was previously recovered from pus specimen of a patient suffering from deep wound infection who was admitted to Ain Shams specialized hospital. This isolate was identified via sequencing the 16S ribosomal RNA and was deposited in the NCBI GenBank (Accession code, MK341125.1) as well as in the Culture Collection Ain Shams University (CCASU) of the World Data Centre for Microorganisms (WDCM) (http://ccinfo.wdcm.org/collection/by_id/1186).

### Animals

Female Wistar rats weighing about 110–120 g were used throughout the experiment. Animals were kept on a 12-h light-dark cycle and a persistent temperature of 22 ± 2 °C, housed (one per cage) and fed on standard diet pellets (El-Nasr, Abu Zaabal, Egypt) comprised about 5% fiber, 20% protein, 3.5% fat, 6.5% ash and have free access to water. This study received an approval from the research ethical committee of Faculty of Pharmacy, Ain Shams University, Egypt (Protocol approval number: ACUC-FP-ASU-Nr. 3). This study was carried out in accordance with Care and Use of Laboratory Animals recommendations and ARRIVE guidelines (https://arriveguidelines.org).

### Preparation of the plain (o/w) emulsion cream

The oily phase of the emulsion was prepared by melting the weighed amounts of the lipid components; stearic acid, soft yellow paraffin, in addition to BHT used as an antioxidant in a beaker mounted in a water bath adjusted at 70 °C. Propylene glycol was then added after complete melting of lipid excipients. The aqueous phase was prepared by dissolving both preservatives (methyl and propyl parabens) in hot deionized water adjusted at 80 °C. Thereafter, the calculated amount of TEA was added. Next, the oily phase was added portion wise to the aqueous phase with continuous homogenization until cooled to room temperature.

### Preparation of the gel formulations

Different gel formers, Carbopol 940, Na alginate, Na CMC, and HPMC E4 were all investigated for the preparation of gel formulae. The least concentration forming gel of each agent was selected depending on tube inversion test (Raghavan and Cipriano 2006). This test was performed by turning a vial containing the sample upside-down (Macosko 1994). The gel used in preparing the emulgel formula (F1) was prepared by dispersing 1% (w/w) Carbopol 940 in deionized water with constant stirring at a moderate speed; then the dispersion was neutralized to pH 6.5–7 using TEA. In emulgel formulations (F2, F3 and F4), the gels were prepared by dispersing 2% (w/w) Na CMC, 3% (w/w) Na alginate and 10% (w/w) HPMC E4, respectively, in deionized water. The prepared colloidal dispersions were then allowed for complete hydration by storing at room temperature for 24 h.

### Preparation of the plain emulgel formulations

The compositions of emulgel formulations are shown in Table 1. The emulgels were prepared by mixing the prepared o/w cream described in “Animals” section with different gel compositions as explained in “Preparation of the plain (o/w) emulsion cream” section at a ratio 1:1 (w/w).

**Table 1:** Composition of different plain emulgel formulations

Ingredients	Concentration (%w/w)			
	F_1_	F_2_	F_3_	F_4_
Stearic acid	7.5	7.5	7.5	7.5
Yellow soft paraffin	5	5	5	5
Propylene Glycol	2.5	2.5	2.5	2.5
BHT	0.05	0.05	0.05	0.05
Methyl paraben	0.18	0.18	0.18	0.18
Propyl paraben	0.02	0.02	0.02	0.02
Triethanolamine	0.675	–	–	–
Carbopol 940	0.5	–	–	–
Na CMC	–	1	–	–
Na alginate	–	–	1.5	–
HPMC E4	–	–	–	5
Water Q.S. to	100	100	100	100

*BHT* Butylated hydroxytoluene, *Na CMC* sodium carboxymethyl cellulose, *HPMC* Hydroxypropyl methyl cellulose

### Preparation of the medicated emulgel formulations

For the preparation of medicated emulgels, the aqueous vehicle in gel formulations was partially or totally replaced by the GE to form either 25 or 50% w/w concentration in emulgel formulae, while two LRJ concentrations (2 and 4% w/w) were incorporated by initially levigating the accurately weighed LRJ amounts in the o/w emulsion cream till a homogenous product was obtained. These medicated emulgels were all employed in the in-vivo studies.

### Characterization of the prepared emulgel formulations

#### Physical examination

The emulgel formulas were examined for various physical parameters including, color, homogeneity, consistency, and phase separation.

#### pH determination

The pH values the prepared emulgels were determined by using a pH meter (Orion, model 420A, USA).

#### Rheological studies

The rheology of the emulgel formulations was examined by using a cone and plate viscometer (Brookfield DV-III ultra-programmable controlled with Brookfield Rheocalc operating software, USA), adjusted at 100 rpm and 25 ± 0.5 °C.

#### Determination of emulgel spreadability

About 100 mg of the emulgel was deposited in the center of a glass plate (5 × 5 cm). Another glass plate of the same dimension was dropped onto the first one from 5 cm. A constant weight (0.5 kg) was added on the glass plate surface and maintained in position for 1 min. The diameter of the spread circle in cm was measured after weight removal (Khullar et al. 2011).

#### Determination of emulgel extrudability

Extrudability is the measurement of emulgel ability to flow from collapsible tubes. The quantity (g) of emulgel extruded from lacquered aluminum collapsible tube after application of a constant weight (500 g) in 10 s was determined. This examination was done in triplicates for each formula. The results of all the formulations were recorded in g/s (Thakur et al. 2012).

### Quantitative determination of RJA and S-allyl cysteine found in LRJ and aqueous GE, respectively

Both RJA and SAC contents in the prepared emulgels were assessed by dissolving a known amount of formulation in methanol–water (50:50) solvent system by sonication. RJA and SAC concentrations were measured after suitable dilution using high performance liquid chromatography (HPLC) technique, as previously reported by Zhou et al. (2007) and Yoon-Ho et al. (2012) with some modifications. The chromatographic system composed of Agilent Technologies 1200 series LC—G1311 equipped with a solvent delivery pump and G1315D diode array detector using a Phenomex-C18 analytical column (5 μm particle size; 250 × 4.6 mm ID) maintained at 25 or 38 °C, for RJA and SAC analysis, respectively. For RJA analysis, the mobile phase was methanol–water (60:40 v/v).

The UV detector was set at 210 nm. While for SAC analysis in the GE, the mobile phase was composed of eluent A consisted of 20 mmol/L sodium dihydrogen phosphate and 10 mmol/L heptane sulfonic acid with water adjusted to pH 2.1 (Arnault et al. 2003). The gradient program used is presented in Table 2. The UV detector was set at 208 nm. The data were measured using ChemStation B.04.01 software (https://www.agilent.com). Both RJA and SAC contents (%) were determined as % of the theoretical amounts and were calculated in triplicate.

**Table 2:** Gradient elution program

Eluent	Time (min)							
	0	5	25	26	28	30	40	50
A	100	70	46	0	0	100	100	100
B	0	30	54	100	100	0	0	0

### In-vitro diffusion studies

Emulgel (0.5 g) was evenly applied onto the surface of a dialysis cellulose membrane (MWCO 12,000–14,000). The dialysis membrane was clamped on a glass cup with a cross-sectional area of 6.2 cm^2^ with a rubber band. The glass cup was then inverted under the surface of 25 mL phosphate buffer (pH 5.5) containing 30% methanol in a closed glass jar. The whole assembly was maintained at 37 ± 0.5 °C in a shaking water bath. Two-milliliters samples were collected at intervals of, 0.5, 1, 1.5, 2, 2.5, 3, 4, 5 and 6 h and immediately replaced with fresh dissolution medium. Samples were filtered with Millipore^®^ disposable syringe filter 0.22 µm (Merck KGaA, Darmstadt, Germany) and then analyzed for drug content by HPLC technique as previously described in “Characterization of the prepared emulgel formulations” section.

### Stability studies

The selected medicated emulgel formulation was stored in amber-glass jars at room temperature and under refrigeration at 5 ± 3 °C for 3 months. After storage, the formula was tested for its physical appearance, pH, rheological behavior, and drug contents.

### Testing the effect of combined LRJ and GE in animal model of MRSA skin infection

The MRSA skin infection rate model was performed according to El-Gayar et al. (2016) with some minor modifications. The first application of any tested formula to the injured skin was 4 h post-infection (dose: 1 mg of the tested agent/1 g of the rat body weight/twice daily) and additional application was repeated twice daily, with 12 h interval, for 10 days (El-Gayar et al. 2016; Tsao et al. 2003; Dai et al. 2010; Aguiar et al. 2009; Kugelberg et al. 2005).

Four control and four test groups, six rats each, were included as follows:

- Group I: control, intact, non-infected, untreated.
- Group II: control, injured, infected, untreated.
- Group III: control, injured, infected, vehicle.
- Group IV: control, injured, non-infected, untreated.
- Group V: injured, infected, treated with Formula 1 (2% LRJ + 25% GE).
- Group VI: injured, infected, treated with Formula 2 (4% LRJ + 25% GE).
- Group VII: injured, infected, treated with Formula 3 (2% LRJ + 50% GE).
- Group VIII: injured, infected, treated with Formula 4 (4% LRJ + 50% GE).

The follow up of the results was carried out by (I): measuring the wound diameter at D1, D3, D7, D10; (II): photographing the wound using digital camera at D9 of injury and, (III): Histopathological examination of the fixed samples at D10. Autopsy samples were taken from rats’ skin, in each group, and fixed in 10% formol for 24 h before examination (Suvarna et al. 2019).

### Statistical analysis

Data are displayed as mean ± S.D. Statistical analysis of wound diameter at different time intervals was performed by two-way analysis of variance (ANOVA) followed by Tukey’s post-hoc test at *P *< 0.001, using GraphPad Instat software, version 3.00 (GraphPad Software, La Jolla, CA, USA).

## Results

### Preparation of different emulgel formulations

The emulgel was prepared by blending o/w emulsion cream with a gel at a ratio of 1:1 w/w. The compositions of different emulgel formulations are presented in Table 1. One of the main ingredients of the emulgel formulation is the gelling agent. Different gel formers, namely, Carbopol 940, Na CMC, Na alginate and HPMC E4 were studied.

### Characterization of the prepared plain and medicated emulgel formulations

The characterization parameters (pH, viscosity, spreadability and extrudability) of the prepared plain and medicated emulgels were determined and the results are collected in Table 3. All prepared plain emulgels were white in color with smooth homogenous consistency. The pH of all emulgels was found to range between 6.7 and 6.9 confirming skin compatibility. As shown in Table 3, the viscosities of the prepared plain emulgels were measured and their values were recorded between 1109 and 2633 cPs. The spreadability and extrudability data of plain formulations can be arranged in descending order as follows: F_3_ = F_2_ > F_1_ > F_4_, as shown in Table 3.

**Table 3:** pH, viscosity, spreadability and extrudability results of plain and medicated emulgel formulations

Emulgel formula code	Mean data^a^ (SD)			
	pH	Viscosity (cps)	Spreadability (cm)	Extrudability (g/s)
F_1_	6.90 (0.02)	1761.33 (141)	3.75 (0.11)	1.02 (0.21)
F_2_	6.85 (0.01)	1109.00 (95)	4.50 (0.05)	1.42 (0.32)
F_3_	6.80 (0.02)	1279.00 (87.02)	4.51 (0.03)	1.40 (0.54)
F_4_	6.70 (0.04)	2633.33 (444.33)	3.30 (0.02)	0.81 (0.43)
MF_1_	ND	ND	ND	ND
MF_2_	ND	ND	ND	ND
MF_3_	6.85 (0.05)	1219.67 (105.77)	4.56 (0.01)	1.36 (0.56)
MF_4_	6.30 (0.05)	2719.33 (413.87)	3.12 (0.03)	0.71 (0.64)

(F1–F_4_) refer to plain emulgels prepared with Carbopol 940, Na CMC, Na alginate and HPMC E4, respectively. (M-F1–M-F_4_) refer to medicated emulgels containing 4% LRJ and 50% GE and prepared with Carbopol 940, Na CMC, Na alginate and HPMC, respectively

*ND* not determined, *SD* standard deviation

^a^Average of three determinations

The light-yellow medicated emulgels were prepared using the highest concentrations of both respective LRJ and aqueous GE, 4 and 50% w/w for characterization. After formulation, a phase separation and complete liquefaction occurred after the inclusion of GE into Na CMC-based emulgel. Moreover, medicated emulgel containing Carbopol exhibited uneven consistency with clumps formation. Hence these formulae were rejected, and their characterization tests were not performed. The medicated emulgels formulated with Na alginate and HPMC exhibited smooth homogeneous consistency with an almost neutral pH values, as obvious in Table 3. Moreover, these medicated preparations showed close viscosity values as their respective plain emulgels. The highest viscosity was pertained to HPMC-containing emulgels either plain or medicated. These formulations appeared sticky in consistency with slight difficulty upon application as confirmed by their low spreadability and extrudability values as presented in Table 3. Na alginate-based medicated emulgel revealed acceptable viscosity, spreadability and extrudability values. Therefore, Na alginate-based emulgel was selected as the optimized medicated product for further in-vitro and in-vivo studies.

### Determination of drug contents

RJA and SAC are of the main active constituents in LRJ and aqueous GE, respectively. The contents of RJA and SAC were thus traced in the optimized Na alginate emulgel formulation using a validated chromatographic assay by HPLC and found to range from 95.76 to 103.23% and from 91.67 to 94.32%, respectively (Table 2).

### In-vitro drug diffusion studies

**Fig. 1:**
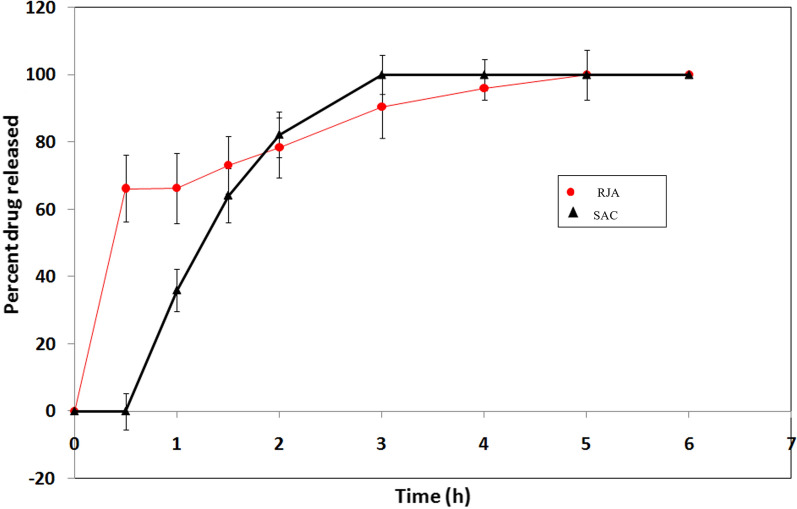
Release profiles of RJA and SAC from Na alginate emulgel formula (MF_3_) across dialysis cellulose membrane

The in-vitro diffusion experiments of both RJA and SAC from the chosen Na alginate emulgel formulation were performed using dialysis membrane technique, and their profiles were then constructed and illustrated in Fig. 1.

The results revealed that the total RJA content present in LRJ was released from emulgel after 5 h with an initial burst release of about 65 ± 9.95% after 30 min only. However, SAC began to release after starting the in-vitro diffusion experiment by 30 min. with a gradual release thereafter reaching 100% after 3 h. Mathematical kinetic modeling of the release profiles of both actives from the selected emulgel formulation was also determined, and their parameters were collected in Table 4. An almost linear relationship with R^2^ = 0.857 was obtained between the amount of SAC released and the square root of time as proposed by the Higuchi theory, while the first-order equation was the most suitable model describing RJA release kinetics (R^2^ = 0.909) from emulgel.

**Table 4:** Mathematical modeling and drug release kinetics of RJA and SAC-containing Na alginate emulgel formulation according to zero-order, first-order and Higuchi equations

Substance	Zero-order	First-order	Higuchi model	Drug release kinetics mechanism
RJA	R2 = 0.620	R2 = 0.909	R2 = 0.855	First-order
SAC	R2 = 0.735	R2 = 0.574	R2 = 0.857	Higuchi

### Stability studies

The selected medicated emulgel (MF_3_) formulation was stored in amber-glass jars at room temperature and in a refrigerator adjusted at 5 ± 3 °C for 3 months. After storage in either condition, no significant change was observed in the physical appearance, pH, viscosity, and drug contents (*P* > 0.05) of the chosen emulgel. Stability data are presented in Table 5.

**Table 5:** Stability data of the selected medicated Na alginate-based emulgel formula (MF3) after storage at room temperature and under refrigeration (5 ± 3 °C) for 3 months

Storage conditions	Physical appearance	Mean data^a^ (SD)			
		pH	Viscosity (cps)	RJA content (%)	SAC content (%)
Initial	Light-yellow smooth homogenous	6.85 (0.05)	1219.67 (105.77)	99.34 (3.52)	95.33 (3.67)
Room temperature	Light-yellow smooth homogenous	6.92 (0.05)	1205.34 (67.58)	97.25 (5.78)	90.88 (5.76)
5 °C ± 3	Light-yellow smooth homogenous	6.80 (0.06)	1245.26 (70.25)	101.45 (3.11)	92.41 (6.89)

MF_3_ refer to medicated Na alginate-based emulgel containing 4% LRJ and 50% GE

*SD* standard deviation

^a^Average of three determinations

### Testing the effect of combined LRJ and GE in animal model of MRSA skin infection

As indicated in Table 6, the skin from uninjured uninfected animals was completely intact (group I). The healing rate of the injured but uninfected skin (group IV) is much more rapid than that seen in the other untreated groups infected with MRSA (groups II and III). At D3, all the injured groups started to heal. At D7, there were further decreases in wound size of treated groups, being more pronounced at groups IV, VII and VIII. While comparing the effectiveness of the four emulgels with different combinations of LRJ and GE at D 10, it was found that the formulas containing 25% of GE (groups V and VI) are generally less effective than the others having 50% GE (groups VII and VIII) that caused significant wound contraction even more than the spontaneous healing of uninfected group (IV). It is noteworthy that 4% LRJ combined with 50% GE (group VIII) had the maximum wound healing properties, being more effective than 2% LRJ and 50% GE (group VII) together with no significant difference from the control uninjured rats (group I).

**Table 6:** The effect of different combinations of LRJ and GE on wound diameter at days 1, 3, 7 10 in an animal model of MRSA skin infection

Days post-induction	Wound diameter (mm)							
	Group I	Group II	Group III	Group IV	Group V	Group VI	Group VII	Group VIII
D1	0	19.7* ± 0.52	19.4* ± 0.83	19.4* ± 0.69	19.5* ± 0.39	19.5* ± 0.51	19.7* ± 0.45	19.4* ± 0.84
D3	0	17.6* ± 0.84	14.2*^,#^ ± 0.67	13.1*,^#^ ± 0.62	18.4* ± 0.87	16.5* ± 0.79	14.5*^,#^ ± 0.69	14.0*^,#^ ± 0.66
D7	0	14.0* ± 0.66	10.4*^,#^ ± 0.49	8.1*^,#^ ± 0.38	14.7* ± 0.7	11.4*^,#^ ± 0.55	4.6*^,#,†^ ± 0.22	3.1*^,#,†^ ± 0.14
D10	0	9.9* ± 0.47	8.5*^,#^ ± 0.41	2.3*^,#^ ± 0.11	5.5*^,#^ ± 0.26	5.1*^,#^ ± 0.24	1.3*^,#,†^ ± 0.06	0^#,†^

Data are presented as Mean ± S.D. n = 6. Statistical analyses were carried out by Two-way ANOVA, followed by Tukey’s post-hoc test. *Significantly different from the corresponding group I (uninjured control) at *P* < 0.001. ^#^Significantly different from the corresponding group II (injured, infected, untreated) at *P* < 0.001. ^†^Significantly different from the corresponding group IV (injured, uninfected, untreated) at *P* < 0.001. Group I: control, intact, non-infected, untreated, Group II: control, injured, infected, untreated, Group III: control, injured, infected, vehicle, Group IV: control, injured, non-infected, untreated, Group V: injured, infected, treated with Formula 1 (2% LRJ + 25% GE), Group VI: injured, infected, treated with Formula 2 (4% LRJ + 25% GE), Group VII: injured, infected, treated with Formula 3 (2% LRJ + 50% GE), Group VIII: injured, infected, treated with Formula 4 (4% LRJ + 50% GE)

**Fig. 2:**
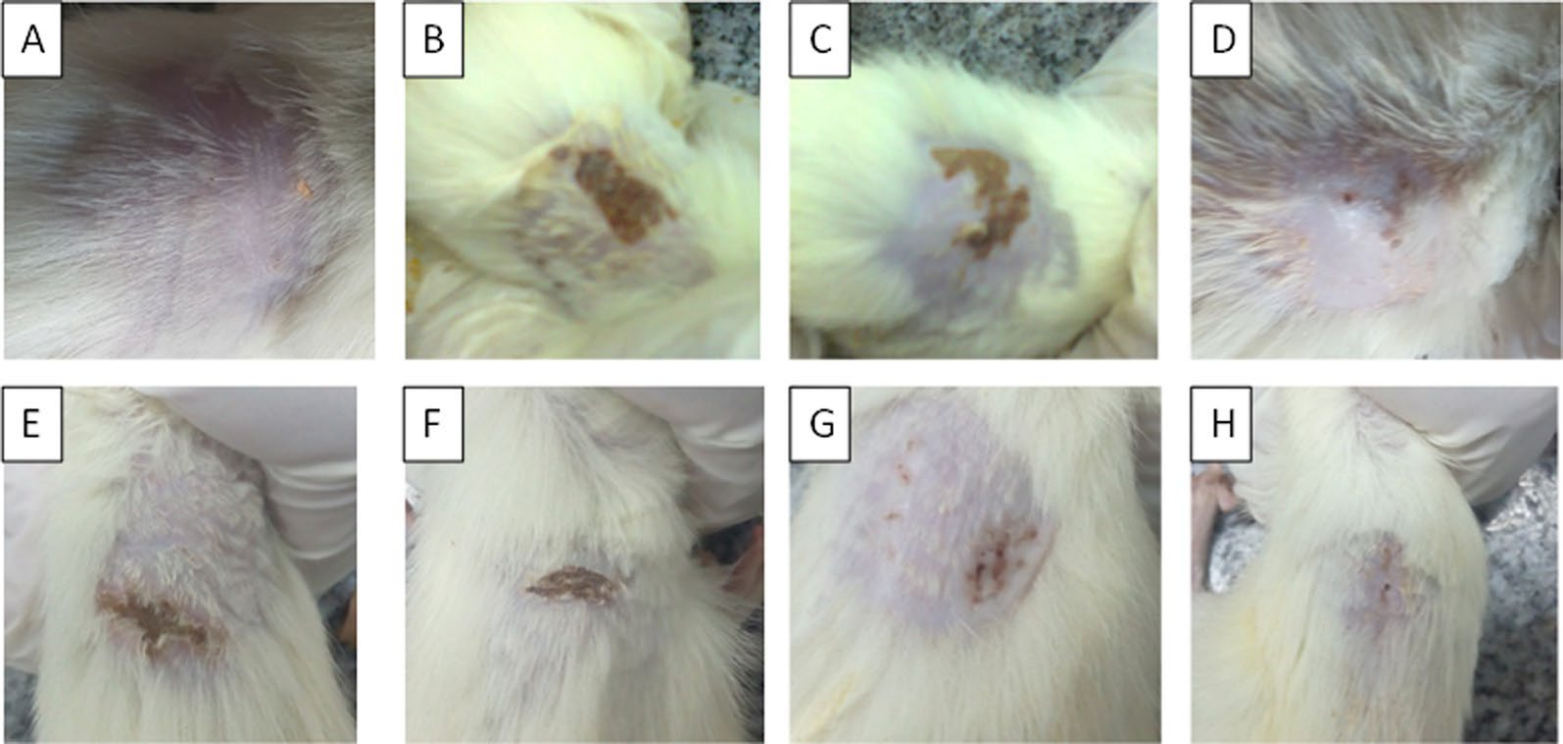
Images of injured skin at 9th day of injury of a representative rat from: **A** control, intact, non-infected, untreated; **B** control, injured, infected, untreated; **C** control, injured, infected, vehicle; **D** control, injured, non-infected, untreated; **E** injured, infected, treated with Formula 1 (2% LRJ + 25% GE); **F** injured, infected, treated with Formula 2 (4% LRJ + 25% GE); **G** injured, infected, treated with Formula 3 (2% LRJ + 50% GE); **H** injured, infected, treated with Formula 4 (4% LRJ + 50% GE)

These findings were in harmony with photographs taken from representative animals from each group at D9 of the experiment, as shown Fig. 2. Interestingly, the wounds in untreated animals infected with MRSA (Fig. 2B, C) were wet (oozing) and showed pus formation when compared to injured uninfected skin (Fig. 2D) that did not show any suppuration. In addition, the degrees of oozing were markedly reduced upon treatment with LRJ and GE (Fig. 2E–H), which were correlated with the efficacy of different formulations. All the previous findings ensured that the used MRSA isolate was pathogenic and caused surgical site infections (SSTIs) in rats.

**Fig. 3:**
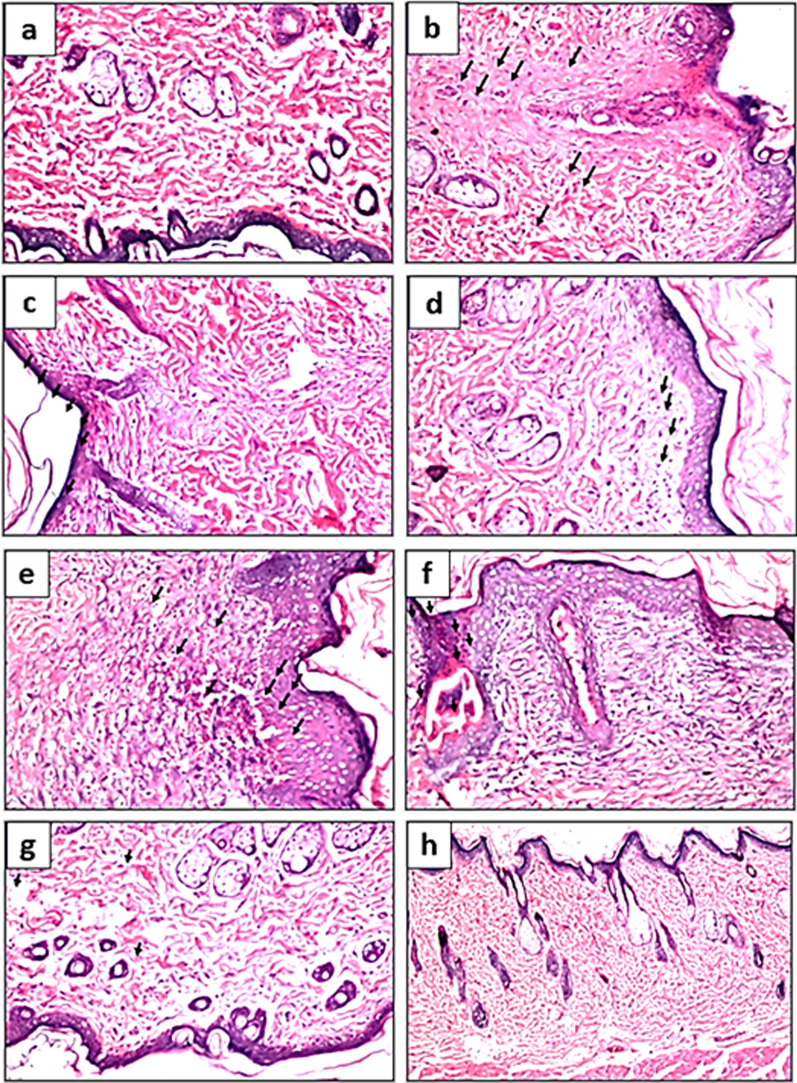
Histopathological examination of rat skin from: **a** group I (No wound) showing the normal histological structure of the epidermis, dermis, subcutaneous tissue and adipose tissue as well as musculature; **b** group II (Wound, Infection, Untreated) showing focal necrosis and hyalinization were detected in the epidermis, dermis and extended to subcutaneous tissue in association with inflammatory cells infiltration and collagen proliferation; **c** group III (Wound, Infection, Vehicle) showing focal atrophy in the epidermis while the underlying dermis showed fibrosis. Focal few inflammatory cells infiltration was detected in the subcutaneous tissue; **d** group IV (Wound, no Infection, Untreated) showing focal fibrosis was detected in the dermis directly underneath the epidermis associated with oedema and few inflammatory cells infiltration in subcutaneous tissue; **e** group V (Wound, Infection, Treated with Formula 1) showing focal acanthosis in the epidermis accompanied by hyalinization in the underlying dermis as well as musculature. The subcutaneous tissue showed fibrosis with inflammatory cells infiltration and edema; **f** group VI (Wound, Infection, Treated with Formula 2) showing focal acanthosis in the epidermis while the underlying dermis had fibrosis. The subcutaneous tissue showed inflammatory cells infiltration and focal hemorrhage; **g** group VII (Wound, Infection, Treated with Formula 3) showing no histopathological alteration in the skin epidermis and dermis while the subcutaneous adipose tissue showed congestion in the blood vessels; **h** group VIII (Wound, Infection, Treated with Formula 4) showing no histopathological alteration in the skin epidermis, dermis, subcutaneous tissue and musculature

The obtained findings were verified by histopathological examinations at the day 10, as displayed in Fig. 3. Wound infection with MRSA resulted in massive damage of skin structure manifested by necrosis, hyalinization, inflammatory cell infiltration and edema (Fig. 3b, c). Wound induction without MRSA infection resulted in mild changes like focal fibrosis and few inflammatory cells infiltration that together could be considered as a normal healing process in any tissue damage (Fig. 3d). It is worth mentioning that massive progression in healing process was revealed in groups VII and VIII (injured, infected, treated with combinations having 50% GE) when compared to other experimental groups having only 25% GE. At day 10, groups VIII and VII were almost completely healed (Fig. 3g, h) while the other groups were still injured with pathological changes such as focal acanthosis in epidermis with fibrosis and hyalinization of the dermis (Fig. 3e, f).

## Discussion

This study was conducted to formulate and test stability and pharmaceutical properties of different emulgel topical preparations containing different concentrations of both LRJ and GE as well as to evaluate their activities to eradicate the topical infections of MRSA using a superficial skin infection animal model. To achieve these goals, different emulgel formulations were prepared. One of the main ingredients of the emulgel is the gelling agent (Carbopol 940, Na CMC, Na alginate and HPMC E4 were studied). As previously known, the concentration of the gel former is of great value regarding drug distribution and product handling (Khan et al. 2013). Therefore, the concentration of the gelling agent was adjusted to the lowest concentration forming gel via the tube inversion test. This test is considered the most common diagnostic test of gelation assuming that a sample having a yield stress (gel) will not flow whereas a viscous but inelastic sample (sol) will show appreciable flow (Sánchez-Cid et al. 2021). Hence, the concentrations of various gelling agents were maintained at 1, 2, 3 and 10% for Carbopol 940, Na CMC, Na alginate and HPMC E4, respectively. These concentrations were considered double the lowest values required, taking into consideration the further twofold dilution of the formed gels when preparing emulgels.

The characterization parameters (pH, viscosity, spreadability and extrudability) of the prepared plain and medicated emulgels were determined. The almost neutral pH of the different plain formulations confirmed the lack from any skin irritation potential, and hence skin compatibility. The viscosity of the emulgel formulations generally reflects its consistency (Khullar et al. 2011). The consistency of emulgel formulations can be ranked according to their viscosity values as follows: F_4_ (HPMC) > F_1_ (Carbopol) > F_3_ (Na alginate) = F_2_ (Na CMC). The spreadability and extrudability of plain formulations were inversely related to the viscosity data of each emulgel formulation (Khullar et al. 2011). Then, the light-yellow medicated emulgels were prepared using the highest concentrations of both respective LRJ and aqueous GE, 4 and 50% w/w. After formulation, a phase separation and complete liquefaction occurred after the inclusion of GE into Na CMC-based emulgel. Similar finding was previously reported with Aloe vera gel (Khan et al. 2013). Moreover, medicated emulgel containing Carbopol showed the formation of visible aggregates and clumps, this could be attributed to TEA present in excess that may interact with acidic components in both actives causing physical incompatibilities manifested in form of clumps. The medicated emulgels formulated with Na alginate and HPMC E4 were considered safe for skin applications with almost neutral pH values. The characteristics of these medicated emulgels were not altered proving that the actives did not have any influence on the physical properties of the formulations.

However, HPMC-based emulgels revealed a kind of stickiness, most probably due to the known adhesive property of the cellulosic derivative polymer (Fahs et al. 2010). The characterization results indicated that Na alginate emulgel can be applied easily with small amount of shear while maintaining a good contact time at the site of application. The contents of RJA and SAC in Na alginate emulgel formulation demonstrated dose uniformity. Moreover, the in-vitro diffusion experiments proved a controlled release of both actives from Na alginate Emulgel with a diffusion-controlled mechanism of SAC release, while RJA release rate was directly proportional to drug amount in the emulgel confirming a first-order kinetics. Being stable, the medicated Na alginate-based emulgel was thus chosen to be employed during the subsequent in vivo studies.

The activity of selected medicated emulgel formulation to eradicate the topical infection of MRSA was evaluated in-vivo using a superficial skin infection rat model. Many studies in the literature concerned with superficial skin infection in mice/rats (Aguiar et al. 2009; Kugelberg et al. 2005; Masson-Meyers et al. 2020; Guay 2003; Avci et al. 2013). To test the effect of the mentioned emulgel formulation, eight groups (four control and four tested groups; (2% LRJ + 25% GE), (4% LRJ + 25% GE), (2% LRJ + 50% GE) and (4% LRJ + 50% GE), The current findings indicated that 4% LRJ combined with 50% GE (group VIII) had the maximum wound healing properties as it was more effective than the combination of 2% LRJ and 50% GE (Group VII). These findings were in line with photographs taken from representative animals from each group at D9 of the experiment. These findings were verified via histopathological examinations at D10 where groups VIII and VII were almost completely healed. However, the other groups were still injured.

It is worth mentioning that wounds in groups treated with the medicated emulgel formulae were dry and no exudates or pus were observed when compared to the infected untreated group (oozing wound). This implies that these emulgels could totally/partially eradicate MRSA. These results agree with Ejaz and coworkers (2003) who found that garlic (*Allium sativum*) has powerful antioxidant and antiseptic effect (Ejaz et al. 2003) and remarkable antimicrobial effect; activities against many MDR Gram positive and Gram-negative pathogens (Iwalokun et al. 2004).

Regarding RJ, the results suggest that RJ may contain active components which have strong bactericidal or bacteriostatic effect as well as healing promoting effect. Some studies had reported that RJ contain active protein “royalisin” with bactericidal activity against Gram positive bacteria, while 10-hydroxy decanoic acid has healing promoting ability and broad-spectrum antibacterial activity (Hornitzky and Smith 1998; Melliou and Chinou 2005; Kwakman et al. 2010; Siavash et al. 2011). Moreover, it was reported that RJ has broad spectrum antibacterial activity due to the presence of trans-10-hydroxydec-2-enoic acid, 3-hydroxydodecanoic acid, 11-oxododecanoic acid, and 11-S-hydroxydodecanoic acid (Melliou and Chinou 2005). However, some peptides and proteins present in RJ have also been shown to possess strong antibacterial properties (Kwakman et al. 2010; Siavash et al. 2011) Because of their, mast cell degranulating activities, together with their broad-spectrum antibacterial properties, make them desirable models for the development of new therapies (Fontana et al. 2004). Some studies attributed its activity to cis-2-decenoic acid, contained in RJ that acts as a dispersant to dissolve protein, nucleic acids, polysaccharides which are the main contents of biofilm (Simuth 2001). In-vitro, some studies suggest that RJ samples were active against the growth of the bacterial strains tested (García et al. 2010).

At the end, it could be deduced that the emulgel formula containing (4% LRJ + 50% GE) has the highest wound healing efficacy, followed by the formula containing (2% LRJ + 50% GE) that showed also high activity. The other two formulas that have 25% GE were much less effective. emulgel formula containing (4% LRJ + 50% GE) has shown the highest wound healing efficacy, followed by the formula containing (2% LRJ + 50% GE) that showed also optimum activity. The other two formulas that have 25% GE were much less effective. Emulgel formula used for treating MRSA infections exhibited both anti-inflammatory activity in addition to their antimicrobial effects (Yang et al. 2018). The anti-inflammatory, antibacterial and antibiofilm properties via of RJ and GE have been previously reported (Yang et al. 2018; Shao et al. 2020). Based on our preclinical study, the emulgel formula containing both LRJ (4%) and GE (50%) displaying remarkable wound healing and antibacterial capacities and therefore, clinical studies should be conducted to ensure its safety and efficacy in human. In conclusion, the physicochemical properties and drug release, the medicated emulgel formulation (MF_3_) prepared with Na alginate was found to be suitable for topical application. Based on the in-vivo testing and current laboratory findings, the emulgel formula containing 4% LRJ and 50% GE has shown the highest wound healing efficacy, followed by the formula containing 2% LRJ and 50% GE that showed also high activity. The other two formulas that have 25% GE were much less effective. Therefore, it could be concluded that GE has a more influence on wound healing than LRJ. In summary, through this study, we believe that the topical application of emulgel formula mentioned above, may be very effective in the treatment of MRSA skin infections and we advised for further clinical studies to confirm their safety and efficacy in human.

## Data Availability

All data generated or analyzed during this study are included in this published article.

## References

[CR1] Aguiar MG, Silva DL, Nunan FA, Nunan EA, Fernandes AP, Ferreira LAM (2009). Combined topical paromomycin and oral miltefosine treatment of mice experimentally infected with *Leishmania* (*Leishmania*) major leads to reduction in both lesion size and systemic parasite burdens. J Antimicrob Chemother.

[CR2] Arnault I, Christidès JP, Mandon N, Haffner T, Kahane R, Auger J (2003). High-performance ion-pair chromatography method for simultaneous analysis of alliin, deoxyalliin, allicin and dipeptide precursors in garlic products using multiple mass spectrometry and UV detection. J Chromatogr.

[CR3] Avci P, Sadasivam M, Gupta A, De Melo WC, Huang Y-Y, Yin R, Chandran R, Kumar R, Otufowora A, Nyame T, Hamblin MR (2013). Animal models of skin disease for drug discovery. Expert Opin Drug Discov.

[CR4] Boukraâ L, Sulaiman SA (2009). Rediscovering the antibiotics of the hive. Recent Pat Anti-Infect Drug Discov.

[CR5] Brudzynski K, Sjaarda C, Lannigan R (2015). MRJP1-containing glycoproteins isolated from honey, a novel antibacterial drug candidate with broad spectrum activity against multi-drug resistant clinical isolates. Front Microbiol.

[CR6] Cong Y, Yang S, Rao X (2019). Vancomycin resistant *Staphylococcus aureus* infections: a review of case updating and clinical features. J Adv Res.

[CR7] Dai T, Huang YY, Sharma SK, Hashmi JT, Kurup DB, Hamblin MR (2010). Topical antimicrobials for burn wound infections. Recent Pat Anti-Infect Drug Discov.

[CR8] Dinkov D, Stratev D, Balkanska R, Sergelidis D, Vashin I (2016). Reduction effect of royal jelly and rape honey alone and in combination against methicillin-resistant *Staphylococcus aureus* (MRSA) strains. J Bacteriol Virol.

[CR9] Donlan RM, Costerton JW (2002). Biofilms: survival mechanisms of clinically relevant microorganisms. Clin Microbiol Rev.

[CR10] Duval RE, Grare M, Demoré B (2019). Fight against antimicrobial resistance: we always need new antibacterials but for right bacteria. Molecules.

[CR11] Ejaz S, Woong LC, Ejaz A (2003). Extract of garlic (*Allium Sativum*) in cancer chemoprevention. Exp Oncol.

[CR12] El-Gayar MH, Aboshanab KM, Aboulwafa MM, Hassouna NA (2016). Antivirulence and wound healing effects of royal jelly and garlic extract for the control of MRSA skin infections. Wound Med.

[CR13] Fahs A, Brogly M, Bistac S, Schmitt M (2010). Hydroxypropyl methylcellulose (HPMC) formulated films: relevance to adhesion and friction surface properties. Carbohydr Polym.

[CR14] Fontana R, Mendes MA, de Souza BM, Konno K, César LMM, Malaspina O, Palma MS (2004). Jelleines: a family of antimicrobial peptides from the Royal Jelly of honeybees (*Apis mellifera*). Peptides.

[CR15] Foster TJ, Geoghegan JA, Ganesh VK, Höök M (2014). Adhesion, invasion and evasion: the many functions of the surface proteins of *Staphylococcus aureus*. Nat Rev Microbiol.

[CR16] Fratianni F, Riccardi R, Spigno P, Ombra MN, Cozzolino A, Tremonte P, Coppola R, Nazzaro F (2016). Biochemical characterization and antimicrobial and antifungal activity of two endemic varieties of garlic (*Allium sativum* L.) of the Campania Region, Southern Italy. J Med Food.

[CR17] García M, Finola M, Marioli J (2010). Antibacterial activity of Royal Jelly against bacteria capable of infecting cutaneous wounds. J Apiproduct Apimed Sci.

[CR18] Girish VM, Liang H, Aguilan JT, Nosanchuk JD, Friedman JM, Nacharaju P (2019). Anti-biofilm activity of garlic extract loaded nanoparticles. Nanomed Nanotechnol Biol Med.

[CR19] Guay DRP (2003). Treatment of bacterial skin and skin structure infections. Expert Opin Pharmacother.

[CR20] Gunaldi O, Daglioglu YK, Tugcu B, Kizilyildirim S, Postalci L, Ofluoglu E, Koksal F (2014). Antibacterial effect of royal jelly for preservation of implant-related spinal infection in rat. Turk Neurosurg.

[CR21] Hornitzky MAZ, Smith L (1998). Procedures for the culture of *Melissococcus pluton* from diseased brood and bulk honey samples. J Apic Res.

[CR22] Iwalokun B, Ogunledun A, Ogbolu D, Bamiro SB, Jimi-Omojola J (2004). In vitro antimicrobial properties of aqueous garlic extract against multidrug-resistant bacteria and *Candida* species from Nigeria. J Med Food.

[CR23] Khan AW, Kotta S, Ansari SH, Sharma RK, Kumar A, Ali J (2013). Formulation development, optimization and evaluation of aloe vera gel for wound healing. Pharmacogn Mag.

[CR24] Khullar R, Saini S, Seth N, Rana AC (2011). Emulgels: a surrogate approach for topically used hydrophobic drugs. Int J Pharm Biol Sci.

[CR25] Kugelberg E, Norström T, Petersen TK, Duvold T, Andersson DI, Hughes D (2005). Establishment of a superficial skin infection model in mice by using *Staphylococcus aureus* and *Streptococcus pyogenes*. Antimicrob Agents Chemother.

[CR26] Kwakman PHS, te Velde AA, de Boer L, Speijer D, Vandenbroucke-Grauls MJC, Zaat SAJ (2010). How honey kills bacteria. FASEB J.

[CR27] Laid B (2015). Bee products: the rediscovered antibiotics. Anti-Infect Agents.

[CR28] Li W-R, Shi Q-S, Liang Q, Huang X-M, Chen Y-B (2014). Antifungal effect and mechanism of garlic oil on *Penicillium funiculosum*. Appl Microbiol Biotechnol.

[CR29] Li G, Ma X, Deng L, Zhao X, Wei Y, Gao Z, Jia J, Xu J, Sun C (2015). Fresh garlic extract enhances the antimicrobial activities of antibiotics on resistant strains in vitro. Jundishapur J Microbiol.

[CR30] Li WR, Shi QS, Dai HQ, Liang Q, Xie XB, Huang XM, Zhao GZ, Zhang LX (2016). Antifungal activity, kinetics and molecular mechanism of action of garlic oil against *Candida albicans*. Sci Rep.

[CR31] Lin Y, Zhang M, Wang L, Lin T, Wang G, Peng J, Su S (2020). The in vitro and in vivo wound-healing effects of royal jelly derived from *Apis mellifera* L. during blossom seasons of *Castanea mollissima* Bl. and *Brassica napus* L. in South China exhibited distinct patterns. BMC Complement Med Ther.

[CR32] Macosko CW (1994) Rheology: principles, measurements, and applications by Christopher W. Macosko, 1 edn. Wiley-VCH, USA. https://www.wiley.com/en-us/Rheology%3A+Principles%2C+Measurements%2C+and+Applications-p-9780471185758. Accessed on 22th Jan 2022.

[CR33] Marra A (2004). Can virulence factors be viable antibacterial targets?. Expert Rev Anti Infect Ther.

[CR34] Masson-Meyers DS, Andrade TAM, Caetano GF, Guimaraes FR, Leite MN, Leite SN, Frade MAC (2020). Experimental models and methods for cutaneous wound healing assessment. Int J Exp Pathol.

[CR35] Melliou E, Chinou I (2005). Chemistry and bioactivity of royal jelly from Greece. J Agric Food Chem.

[CR36] Nidadavolu P, Amor W, Tran PL, Dertien J, Colmer-Hamood JA, Hamood AN (2012). Garlic ointment inhibits biofilm formation by bacterial pathogens from burn wounds. J Med Microbiol.

[CR37] Raghavan SR, Cipriano BH, Weiss RG, Terech P (2006). Gel formation: phase diagrams using tabletop rheology and calorimetry. Molecular gels: materials with self-assembled fibrillar networks.

[CR38] Reardon S (2014). WHO warns against “post-antibiotic” era. Nature.

[CR39] Sánchez-Cid P, Jiménez-Rosado M, Alonso-González M, Romero A, Perez-Puyana V (2021). Applied rheology as tool for the assessment of Chitosan hydrogels for regenerative medicine. Polymers (basel).

[CR40] Shao X, Sun C, Tang X, Zhang X, Han D, Liang S, Qu R, Hui X, Shan Y, Hu L, Fang H, Zhang H, Wu X, Chen C (2020). Anti-inflammatory and intestinal microbiota modulation properties of Jinxiang Garlic (*Allium sativum* L.) polysaccharides toward dextran sodium sulfate-induced colitis. J Agric Food Chem.

[CR41] Siavash M, Shokri S, Haghighi S, Mohammadi M, Shahtalebi MA, Farajzadehgan Z (2011). The efficacy of topical Royal Jelly on diabetic foot ulcers healing: a case series. J Res Med Sci off J Isfahan Univ Med Sci.

[CR42] Siddiqui AH, Koirala J (2021) Methicillin resistant *Staphylococcus aureus*. In: StatPearls. StatPearls Publishing, Treasure Island (FL), USA; 2021. http://www.ncbi.nlm.nih.gov/books/NBK482221/. Accessed on 22th Jan 2022. 29489200

[CR43] Simuth J (2001). Some properties of the main protein of honeybee (*Apis mellifera*) royal jelly. Apidologie.

[CR44] Suvarna SK, Christopher L, Bancroft JD (2019). Bancroft's theory and practice of histological techniques.

[CR45] Thakur NK, Bharti P, Mahant S, Rao R (2012). Formulation and characterization of benzoyl peroxide Gellified emulsions. Sci Pharm.

[CR46] Tong SY, Nelson J, Paterson DL, Fowler VG, Howden BP, Cheng AC, Chatfield M, Lipman J, Van Hal S, O'Sullivan M, Robinson JO, Yahav D, Lye D, Davis JS (2016). CAMERA2-combination antibiotic therapy for methicillin-resistant *Staphylococcus aureus* infection: study protocol for a randomised controlled trial. Trials.

[CR47] Tsao S, Hsu C, Yin M (2003). Garlic extract and two diallyl sulphides inhibit methicillin-resistant *Staphylococcus aureus* infection in BALB/cA mice. J Antimicrob Chemother.

[CR48] Turner NA, Sharma-Kuinkel BK, Maskarinec SA, Eichenberger EM, Shah PP, Carugati M, Holland TL, Fowler VG (2019). Methicillin-resistant *Staphylococcus aureus*: an overview of basic and clinical research. Nat Rev Microbiol.

[CR49] Yadav S, Trivedi NA, Bhatt JD (2015). Antimicrobial activity of fresh garlic juice: an in vitro study. AYU.

[CR50] Yang YC, Chou WM, Widowati DA, Lin IP, Peng CC (2018). 10-hydroxy-2-decenoic acid of royal jelly exhibits bactericide and anti-inflammatory activity in human colon cancer cells. BMC Complement Altern Med.

[CR51] Yoon-Ho B, Dao CT, Jae-Hyun L, Mi-Hee W, Jae-Sue C, Byung-Sun M (2012). Quantitative analysis of bioactive compounds in the fruits of *Crataegus pinnatifida* by high-performance liquid chromatography. Nat Prod Sci.

[CR52] Zhou J, Shen J, Xue X, Zhao J, Li Y, Zhang J, Zhang S (2007). Simultaneous determination of nitroimidazole residues in honey samples by high-performance liquid chromatography with ultraviolet detection. J AOAC Int.

[CR53] Zhu X-Y, Zeng Y-R (2020). Garlic extract in prosthesis-related infections: a literature review. J Int Med Res.

